# Comparing drift-diffusion modeling and finger tracking as a window into decision-making in handball penalty situations

**DOI:** 10.1007/s00221-026-07324-8

**Published:** 2026-06-05

**Authors:** Henrietta Weinberg, Florian Müller, Rouwen Cañal-Bruland

**Affiliations:** Department for the Psychology of Human Movement and Sport, Institute of Sport Science, Friedrich Schiller University Jena, Seidelstraße 20, 07749 Jena, Germany

**Keywords:** Evidence accumulation, DDM, Movement trajectories, Anticipation, Sport

## Abstract

Deciding between multiple options in a split second is a crucial aspect in various domains, including traffic, aviation, policing, and sports. Both drift–diffusion modeling (DDM), a computational model that approaches decision-making as noisy evidence accumulation, and finger tracking have been suggested to capture the evolution of a decision over time. In this study, we comparatively applied DDM and finger tracking to examine the processes underlying split-second decision-making within an anticipatory handball penalty task. Participants were shown temporally occluded videos of handball penalties and predicted shot direction by either pointing or continuously swiping toward one of two target areas. We extended previous research by using an optical motion capture system to track trajectories of both pointing and swiping and also calculated drift–diffusion models grouped by response modality. Results indicate that the DDM robustly mirrors the decision-making process. The model reflects the movement differences between pointing and swiping accurately in the non-decision time and shows consistent correlations between response modalities. In contrast, the finger tracking parameters (i.e., area under the curve, velocity, x-flips, and entropy) did not show consistent correlations between pointing and swiping trials and were strongly dependent on response modality. Furthermore, the effect of the response modality manipulation could not be clearly identified by finger tracking parameters. We conclude that DDM when compared to finger tracking seems to provide more modality-invariant insights into the processes underlying decision-making across different response tasks (i.e., modalities).

Decision-making is an omnipresent aspect of everyday life, ranging from trivial tasks such as deciding whether to make tea or coffee in the morning to making significant choices for oneself or others, such as stopping a child aiming to cross the road despite a red stop light. At its core, decision-making is the selection of an action from multiple consequential alternatives (Raab et al. 2019). The field of sport is a particularly fascinating and relevant domain because split-second decisions are abundant and crucial and such decisions are decisive for winning or losing competitions. Due to high time constraints of sports games, adaptive and well-timed decisions, such as deciding whom to block in basketball or selecting where to shoot a penalty in soccer (Noël et al. 2015), are crucial aspects of successful performance in sports (Loffing and Cañal-Bruland 2017). For example, goalkeepers in handball facing a 7 m penalty throw only have a temporal window of approximately 350 ms from ball release until the ball crosses the goal line (Schorer 2007). Within this very short time frame, the keeper has to perceive, decide, and implement a choice that might well make the difference between winning or losing a game. It is well established that success in such time-constrained decision-making situations requires the ability to predict an action’s outcome, that is, to anticipate the likely unfolding of events (Loffing and Cañal-Bruland 2017; Williams and Jackson, 2019). Therefore, the predictions shape and constrain the decision-making process as it evolves and thus, anticipation is an integral component of decision-making under time constraints (e.g., Seidel-Marzi and Cañal-Bruland 2025).

Over the past decades, indeed a large body of research in the field of decision-making and anticipation has emerged (Johnson 2025; Loffing and Cañal-Bruland 2017, for an overview). On the one hand, this literature indicates that, for instance, efficient visual search behavior leads to more effective extraction of information, as demonstrated by higher accuracy scores achieved by experts compared to novices (Savelsbergh et al. 2002; Williams and Jackson, 2019). On the other hand, contextual information (e.g., action preferences of opponents, current game scores, previous outcome sequences, court position; Cañal-Bruland and Mann 2015) is continuously integrated to narrow down the range of likely action outcomes (see Helm et al. 2020, for a Bayesian integration approach in anticipation) and to allow adequate responding under tight temporal constraints and the corresponding levels of uncertainty (Gray and Cañal-Bruland 2018; Gredin et al. 2023; Helm et al. 2020). As concerns contextual information, research has repeatedly shown that congruent contextual and kinematic information (i.e., both point in the same direction) lead to faster response times and higher accuracy scores (Mann et al. 2014; Runswick et al. 2019; for moderation effects by expertise and information certainty, see Jackson et al. 2020; Magnaguagno et al. 2022).

## Continuous approaches to anticipation and decision-making

A potential limitation in the research field of anticipation though is that it has predominantly relied on single end point measures such as response times and accuracy scores. While these measures undoubtedly offer important information about the final outcome of the decision-making process, focusing on just one point in time neglects and may severely limit insights into the dynamic and continuous processes that precede and lead up to the final outcome (i.e., button press left vs. right). Although more advanced methods, such as Bayesian integration have been successfully applied and helped identify distinct influencing factors of the anticipation process (Helm et al. 2020), thus far they have relied on single (end)point choices. Yet, this focus has not gone unnoticed, and there is growing awareness of the potential need to capture the dynamic unfolding of anticipation by means of continuous measures, such as sequential sampling models (Becchio et al. 2018; Gold and Stocker 2017; Smith et al. 2022).

Sequential sampling approaches such as the drift diffusion model (DDM; Ratcliff and McKoon 2008) conceptualize decision-making as noisy evidence accumulation over time. The DDM retroactively infers the likely unfolding of cognitive processes during decision-making from classical endpoint measures, such as accuracy and response times (Forstmann et al. 2016; Gold and Stocker 2017). The DDM represents distinct components of the decision-making process in a two-forced-choice paradigm with four parameters (Ratcliff et al. 2016). Specifically, the drift rate (v) represents the speed of evidence accumulation over time, whereas the separation of the decision boundaries (a) represents the amount of evidence required to arrive at one or the other decision (i.e., a liberal decision style reduces while a conservative decision increases boundary separation). Additionally, the starting point (z) represents the initial state of evidence accumulation, that is, it captures a possible bias for one of the two response alternatives. Finally, the non-decision time (t) captures all processes prior and subsequent to evidence accumulation, including perceptual encoding of the stimulus as well as motor preparation and execution processes that according to DDM assumptions take place before and after decision making proper. Differences in non-decision time are expected to reflect modality-specific motor demands, such as action initiation, movement dynamics as well as constraints. This computational modeling approach towards the investigation of decision-making over time is widespread in neuroscience and cognitive psychology (Myers et al. 2022; Ratcliff et al. 2016).

In recent years, first studies have started to apply DDM to examine decision making and corresponding motor responses, for instance, by comparing different response modalities (Gomez et al. 2015), studying sensorimotor tasks (Carsten et al. 2023), distinct grasping movements (Koul et al. 2019; Quarona et al. 2020), vigilance (Zhong et al. 2025), motor experience (Yan et al. 2025), and contextual information integration in handball (Weinberg et al. 2025). For instance, He and colleagues (2025) investigated the effect of deceptive actions on experts and novices in soccer. Participants watched occluded videos of a 1-vs.-1 soccer dribbling scenario with or without a deceptive action (i.e., step-over move) and had to decide about the final movement direction (i.e., left or right) via key press. Using DDM, their results indicated a significantly higher drift rate, narrower decision boundaries as well as a shorter non-decision time (i.e., less time for perceptual and motor processes) and higher starting point for genuine compared to deceptive trials. Based on those findings, they concluded that deceptive trials are more cognitively challenging, as reflected by a slower rate of evidence accumulation, and require more time for perception and motor responses.

Despite its promising applicability and merits, however, DDM is not the only possibility to capture the time course of decision making. Continuously tracking movements during effectuating one’s decision offers another approach frequently adopted in psychological research (Freeman 2018; Maldonado et al. 2019; Schoemann et al. 2021). In general, movement tracking experiments combine a forced choice task with continuous recording of participants’ finger or mouse movement from a starting area towards one of the two target areas, representing two alternative choices. Researchers then analyze deviations from the ideal movement path to trace the evolution of the decision process towards one of the two alternative response options (Schoemann et al. 2021). Consequently, variables such as the area under the curve (AUC; area between the actual and the shortest, i.e., straight, movement path), x-flips (horizontal movement changes), or entropy (movement complexity) are at the heart of movement tracking analyses (see Wirth et al. 2020, for a comprehensive overview). Due to its promise to capture the unfolding of decision making across single trials (Dotan et al. 2019; Koenig-Robert et al. 2024; Smith et al. 2022) motion tracking has seen widespread adoption in research in neurophysiology (Cisek and Kalaska 2010), social categorization (Stolier and Freeman 2016), attention (Xiao et al. 2023), deception (Tabatabaeian et al. 2015), decision making (McKinstry et al. 2008; Vogel et al. 2018), and motor planning (Zhang et al. 2024).

## Comparing DDM and movement tracking

Both DDM and movement tracking are utilized to investigate the decision-making process. However, they are commonly used independently of each other. Naturally, if both methods – sequential sampling approaches such as DDM and continuous movement tracking such as finger or mouse tracking – argue to capture the evolution of decision making over time, one would expect that their findings regarding the decision-making process should converge, that is, yield similar results. However, to date only very few studies have directly compared DDM and movement tracking analysis. As a case in point, Leontyev and Yamauchi (2021) investigated whether the trajectory features of movement tracking correlate with parameters of the DDM (Ratcliff and McKoon 2008). In their experiment, participants completed two different decision-making tasks, each in a keypress and in a mouse-tracking version. After finding high correlations between drift rates and non-decision times of keypress and tracking trials, they subsequently correlated movement trajectory features with the DDM parameters revealing linear relationships between DDM (i.e., boundary separation, non-decision time) and tracking (i.e., maximum velocity, AUC) parameters. DDM parameter drift rate was related to the AUC, calculated as the area between the ideal and the observed trajectory (Wulff et al. 2021) and interpreted as a metric of uncertainty and decision conflict. Previous neurological research (Singh et al. 2021) indicates that, in the context of conflict tasks, velocity exerts an influence on sensory integration which might correspond to the non-decision time of the DDM. In line with this assumption, they employed the maximum velocity to assess impulsivity, and it was found to be negatively correlated with the non-decision time of stop trials in a stop-signal task. Note though, that only the trajectories from the mouse-tracking (that is, across different cognitive tasks) were incorporated in their analysis. Leontyev and Yamauchi (2021) concluded that the response modalities are interchangeable concerning their use in cognitive tasks and that the parameters of tracking and modeling seem to reflect the same underlying cognitive processes.

Adopting a similar design as Leontyev and Yamauchi (2021), Weinberg and colleagues (2025) recently assessed decision making in a sport specific scenario in two experiments. Specifically, participants were shown a series of 7 m handball penalty throws from the goalkeeper’s perspective that were visually occluded at the moment of ball release. Participants predicted the throwing direction of the ball (left vs. right) by either pointing or swiping (response modality conditions) on a touchscreen tablet towards the corresponding target area. The two response modalities allowed both modeling of the decision process (DDM) based on the endpoints as well as movement tracking analysis (swiping trials only).

In the first experiment, a comparison of DDM parameters across pointing and swiping movements validated DDM’s applicability for the handball anticipation task. The second experiment incorporated contextual probability information, showing significant effects not only on the starting point but also on drift rate and non-decision time. In addition to the effect of response modality on non-decision time (Gomez et al. 2015), findings replicated correlations of DDM parameters (i.e., drift rates and non-decision times) between pointing and swiping trials (Leontyev and Yamauchi 2021). However, in contrast to Leontyev and Yamauchi (2021), Weinberg and colleagues reported a differentiated pattern between DDM and tracking parameters: while drift rate showed no significant correlation with tracking parameters, the non-decision time was related to AUC and velocity across both experiments (x-flips and entropy only correlated in one experiment). The results raise the question whether DDM and continuous movement tracking provide similar insights into the decision-making process. An additional shortcoming of both studies (Leontyev and Yamauchi 2021; Weinberg et al. 2025) is that movement tracking data and corresponding analyses were only available for the movement tracking version of the respective task, not allowing the authors to compare DDM and finger tracking with each other.

Taken together, both DDM and movement tracking are used to investigate the temporal dynamics of the decision-making process. However, these approaches are commonly utilized in isolation, and few studies directly examined whether they provide complementary information about the same underlying cognitive processes.

## The current study

To examine if both DDM and movement tracking provide comparable insights into decision-making under uncertainty, the aim of our study is twofold: First, we aim to analyze and replicate the influence of response modality, i.e., pointing and swiping, on DDM parameters in the anticipation task of Weinberg et al. (2025). Second, we aim to extend previous research (i.e., Leontyev and Yamauchi 2021; Weinberg et al. 2025) by examining the relationship between DDM and tracking parameters independently of response modality, thereby ensuring a comprehensive comparison between pointing and swiping trials. To this end, we built on Weinberg et al. (2025) but additionally tracked participants movement during pointing trials via motion capture. Again, the anticipatory handball penalty task incorporated high degrees of uncertainty and conflicting information (e.g., Cañal-Bruland et al. 2015; Helm et al. 2020; Müller et al. 2024) as well as time pressure by requiring the response to be given at the right time and place (Loffing and Cañal-Bruland 2017). As previously discussed, the decision-making process is hypothesized to be reflected in specific tracking parameters on the one hand (Hehman et al. 2015; Song and Nakayama 2009), and in the respective parameters of the DDM on the other hand. In the present task, the demands of perceptual encoding are identical between response modalities, therefore, potential differences in non-decision time are expected to reflect differences in response modality. While pointing is a free movement toward a target area, swiping requires continuous movement with sustained touchscreen contact. Those differences in movement are therefore expected to be expressed in the non-decision time rather than in other DDM parameters. However, tracking parameters are assumed to reflect aspects of the decision-making process as it unfolds into action. If finger tracking mainly captures decision-related components, a relationship across response modalities as well as with DDM parameters ought to be expected. Conversely, a strong response modality dependence would suggest an integrated decision-action dynamic rather than modality-invariant cognitive processes.

In line with Leontyev and Yamauchi (2021), we predict a relationship between DDM (i.e., drift-rate, non-decision time) and movement tracking parameters (i.e., AUC, maximum velocity). In addition, Weinberg et al. (2025) investigated x-flips and entropy as measures of trajectory disorder, which can be utilized as complexity indices. X-flips denote the number of directional changes along the x-axis, whereas entropy signifies spatiotemporal disorder (Hehman et al. 2015; Wulff et al. 2021). It has been demonstrated that both parameters mirror response conflict, whereas higher cognitive conflict results in higher complexity (Wulff et al. 2021). In this regard, we draft three main hypotheses. First, in line with the idea that the decision process unfolds similarly—regardless of response modality—we expect positive correlations of DDM parameters across response modalities, replicating established findings (Leontyev and Yamauchi 2021; Weinberg et al. 2025). Second, if motion tracking analysis represents an equally robust measure of decision making over time, positive correlations of tracking parameters (AUC, x-flips, velocity, entropy) should emerge across both response modalities. Finally, if DDM and motion tracking are both valid measures of the same decision-making process, these measures’ parameters should behave similarly. In detail, we hypothesize that a higher rate of evidence accumulation corresponds with a smaller AUC, fewer x-flips, smaller entropy and higher velocity, and that a higher rate of non-decision time corresponds with a greater AUC, more x-flips, higher entropy and lower velocity. If the decisions reflect the same underlying processes, then the parameters should correlate irrespective of response modality. Furthermore, based on Leontyev and Yamauchi (2021) we predicted a negative relationship between boundary separation and velocity. Lastly, we also analyzed the relationship between non-decision time and tracking parameters based on the physical demands of the movement for which Weinberg and colleagues (2025) reported high correlations in swiping trials.

## Method

### Participants

A total of 29 right-handed participants took part in the experiment. According to a priori power analysis, calculated with G*Power (Faul et al. 2009, Version 3.1.9.7), a total sample size of 23 (power = 0.8, alpha = 0.05, tested one-sided) was required to detect a positive correlation of the DDM parameters of at least *r* = 0.5. Data of two participants had to be removed due to either technical issues or failure to follow instructions, resulting in a final sample of 27 participants (sex: 15 female, 12 male; age: *M* = 20.56 *SD* = 1.95, range = 18–27). Four out of 27 participants reported previous handball experience (*M* = 4.5 years, *SD* = 3.87, range = 1–10) on club and district league level.^1^ The study was part of a research program that was approved by the Ethics Committee of the Faculty of Social and Behavioral Sciences at the Friedrich-Schiller-University Jena (approval number FSV 22/078). All participants provided written informed consent.

### Materials and measures

All experimental instructions and stimuli were presented using standard web technologies (i.e., HTML + Javascript) using the Safari web browser running on a 2018 iPad Pro with a 12.9-inch display on iOS 12.1.3. During the swiping task, the tablet’s touchscreen recorded participants finger movement at a sampling rate of 120 Hz. For motion tracking of participants’ index finger during the pointing task, we used an OptiTrack system with four Prime 17W cameras (1.7 MP resolution, 360 FPS, approx. 2.8 ms latency, 3D accuracy of the Prime series range between approx. 0.1 and 0.3 mm) and the OptiTrack Motive Software (Version 3.0.1) with motion recording triggered on a trial-by-trial basis at a sampling rate of 120 Hz. To this end, a solid white square was shown during the runtime of each trial in the lower left corner of the tablet (vs. solid black between trials). A Raspberry Pi Pico microcontroller (www.raspberrypi.com) captured these trial-by-trial changes in brightness with a photodiode (Velleman VMA407) and then sent a 3.3V TTL signal via its GPIO pins to the TTL sync input of an Optitrack eSync 2 to start and stop Optitrack recording for each trial. For comparability with the touchscreen data, the 3D capture space was calibrated so that its “floor” (x–y dimension) was exactly aligned with the tablet’s screen surface. To arrive at structurally similar movement data to the swiping trials, movement along the vertical (z dimension) was discarded. Although movement trajectories were recorded using different systems, both response modalities were sampled at the same temporal resolution and were subsequently transformed into a common reference frame and resampled to a standardized temporal scale (see Data Analysis). While display and input latencies between tablet and motion capture devices differ, those latencies were stable within response modalities and across trials. Due to identical stimuli across response modalities as well as analyses that require distributions instead of absolute timing, device-specific latency is unlikely to influence results. Video clips of successful 7-m handball penalties, temporally occluded at the moment of ball release, served as stimuli (for details of the acquisition of the videos see Weinberg et al. 2025). We used 50 clips of one player with a mean runtime of 1104.3 ms (*SD* = 164.37, range: 896–1621). An exit questionnaire contained questions on demographic information (age, sex, handedness), visual impairments including the use of aids, and previous experience in handball and other ball sports (professional level, training age).

### Procedure and design

**Fig. 1:**
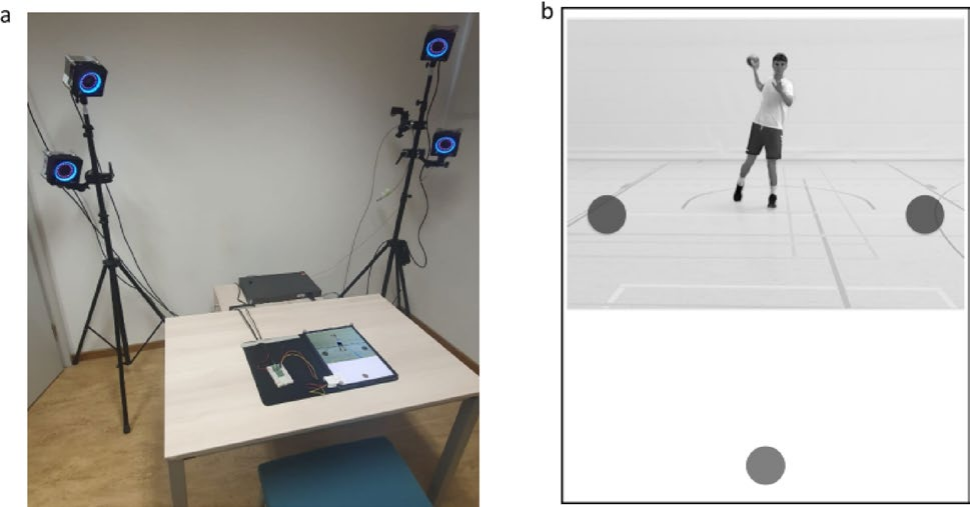
Experimental setup. *Note* Experimental setup with four OptiTrack cameras and a tablet (**a**) as well as a schematic illustration of the task setting displayed on the tablet (**b**)

Participants were first given general information about the procedure. They were then seated at a table with the tablet and in front of four OptiTrack cameras (Fig. 1a). The tablet was used to deliver all instructions and stimuli. Participants were also given a finger sleeve with an OptiTrack marker for their right index finger.

Their task was to complete a series of forced-choice trials in which they predicted the direction of a penalty throw (left vs. right) shown in a short video clip (Fig. 1b). The response method alternated between experimental blocks, with participants indicating the predicted throw direction by either pointing or swiping, constituting the within-subject factor *response modality* (pointing, swiping). In the pointing condition, participants indicated the decision by touching either the left or the right target area with the dominant index finger. In the swiping condition, participants had to make a continuous finger movement from the start area toward the selected target area while maintaining contact with the touchscreen. Each modality block contained the same set of 50 video clips, presented in random order for each participant. In total four blocks were presented (i.e., 100 trials per response modality in total) and block sequence was counterbalanced between participants. According to the DDM literature (Lerche et al. 2017; Voss et al. 2013; Wiecki et al. 2013) those trial numbers are sufficient for fitting models which estimate core parameters. Prior to each experimental block, participants completed 10 familiarization trials with a different set of stimuli from another thrower and were provided feedback about their decision. Each individual trial started with participants touching the starting area at the bottom of the screen with their index finger, which started the video clip. In pointing trials, participants then had to tap one of the two target areas at the top of the screen. In swiping trials, they had to swipe their finger continuously along the screen to the selected target area instead. Instructions were given to time the response as if the ball were to be intercepted at the anticipated location (i.e., at the right time at the right place). That instruction intended to reflect the actual demands of a real-world handball penalty situation, where successful performance depends on both selecting the correct place and executing the movement at the correct time (Schorer 2007). Feedback on errors was provided during familiarization trials but not in experimental trials.

### Data analysis

We performed three levels of analysis. First, the distributions of accuracy scores and response times of decision towards the left and the right direction were used to model the DDM parameters using the python HDDM package (version 0.9.8RC) by Wiecki et al. (2013), following the dockerHDDM workflow by Pan et al. (2025). Response time was defined as the time from stimulus onset (i.e., video onset) until the response was registered in one of the target areas for the first time in both response modalities. In pointing trials, that corresponds to the first touch within the target area, while in swiping trials it is the first entry of the finger into the target region. MCMC sampling was performed with four chains and 20,000 iterations per chain. The first 5000 iterations of each chain were discarded as burn-in, no thinning was applied. Default HDDM priors were used for all parameters. We estimated five models (Table 1). Model convergence was assessed utilizing Gelman-Rubin statistics (R-hat) and effective sample size (ESS). Analyses in the current study are all based on group-level parameters. For the group-level parameters, R-hat values ranged from 1.00 to 1.01, bulk ESS from 399 to 53,937 and tail ESS from 904 to 42,841.^2^ Model comparison was conducted using Deviance Information Criterion (DIC). DIC values were computed after convergences were evaluated and were compared among models that showed overall acceptable convergence according to the recommended criteria (R-hat < 1.01, ESS > 400; Pan et al. 2025). Lower DIC values were interpreted as indicating a better relative model fit. The model that let all four parameters (i.e., drift rate, boundary separation, starting point, non-decision time) vary by response modality (M4) fit the data best, as indicated by the lowest deviance information criterion. Furthermore, we fixed intertrial variability to improve parameter estimation (Lerche and Voss 2016) and calculated posteriors of drift rate, boundary separation, starting point, and non-decision time for pointing and swiping trials and considered an overlap of less than five percent of the posterior proportions to be significant.

**Table 1:** Deviance information criterion of the computed HDDM’s

Model	Model parameter dependencies	DIC values
M0	None	9104.3^1^
M1	Drift rate depends on mode	10,682.7^1^
M2	Non-decision time depends on mode	6717.2
M3	Drift rate and non-decision time depend on mode	6774.1
M4	All four parameters depend on mode	6610.4

*DIC* Deviance information criterion, lower values indicate better model fit; ^1^M0 and M1 showed insufficient convergence for some parameters. Their DIC values are reported for completeness only and were not used for model comparison or inference

Second, we analyzed the trajectory data in R (R Core Team 2023, Version 4.4.3) using the Wulff et al. (2021) mousetrap package (Version 3.2.3). For swiping trials trajectories of finger movement recorded by the touchscreen were readily available. In contrast, finger trajectories from pointing trials were recorded with the motion capture system and constitute 3D data. To allow for comparison with the 2D swiping data, movement data were projected on the tablet’s screen plane and scaled to the same coordinate system. Both recording systems operated at the same sampling rate (120 Hz) and were synchronized at the trial level. Given that analyses in our study focused on aggregated trajectory measures timing offsets at millisecond level are unlikely to have an effect on the results.

The analysis of the tracking data followed the recommendations by Wulff and colleagues (2021). To ensure comparability across trials and response modalities, the movement trajectories were transformed into a common reference frame by mirroring them to one side and aligning them to a standardized start and end point. To account for differences in movement duration and path length, trajectories were resampled to a common temporal resolution using interpolation. No explicit spatial or temporal smoothing or filtering was applied beyond the interpolation-based resampling. After that, we examined whether preprocessing yielded consistent and interpretable trajectory data within each response modality. They showed plausible start and end alignments as well as distributions of kinematic measures, which suggested that preprocessing did not introduce systematic artefacts. Moreover, basic kinematic measures (i.e., RT, maximum acceleration) showed systematic relationships across participants, indicating that the preprocessing preserved meaningful trajectory information while allowing modality-specific movement characteristics (i.e., total distance, y-flips) to remain. Therefore, we computed the area under the curve, maximum velocity, x-flips, and entropy (see Wirth et al. 2020, for a comprehensive overview of the measures). The area under the curve (AUC) quantifies the deviation of the trajectory from the optimal (i.e., straight) path, x-flips represents the number of changes on the horizontal movement axis and entropy captures the irregularity and complexity of the trajectory. Maximum velocity was computed from the resampled trajectories and therefore can be interpreted as relative indicator of movement dynamics. Correlation analyses were used to test for relationships of parameters of each model across tasks, as well as for correlations between corresponding parameters from both models. Multiple regressions were employed to test the relationship of DDM to motion tracking parameters. In the case of directional hypotheses, one-sided testing was used. All analyses were Holm-Bonferroni corrected.

## Results

Response modality (i.e., pointing and swiping) had a significant effect on response times, *t*(26) = 8.70, *p* < 0.001, d = 1.65, and accuracy scores, *t*(26) = 6.10, *p* < 0.001, d = 1.06, with slower but more accurate responses in pointing (*M*_*RT*_ = 1.81 s, *SD*_*RT*_ = 0.33 s; *M*_*acc*_ = 84.0, *SD*_*acc*_ = 8.87) compared to swiping trials (*M*_*RT*_ = 1.30 s, *SD*_*RT*_ = 0.29 s; *M*_*acc*_ = 72.8, *SD*_*acc*_ = 12.0). Subsequent correlation analysis across response modalities showed significant positive relationships between response times, *r*(25) = 0.52, *p* = 0.018, and accuracies, *r*(25) = 0.62, *p* = 0.002, of pointing and swiping trials.

### Effects of response modality on DDM parameters

**Fig. 2:**
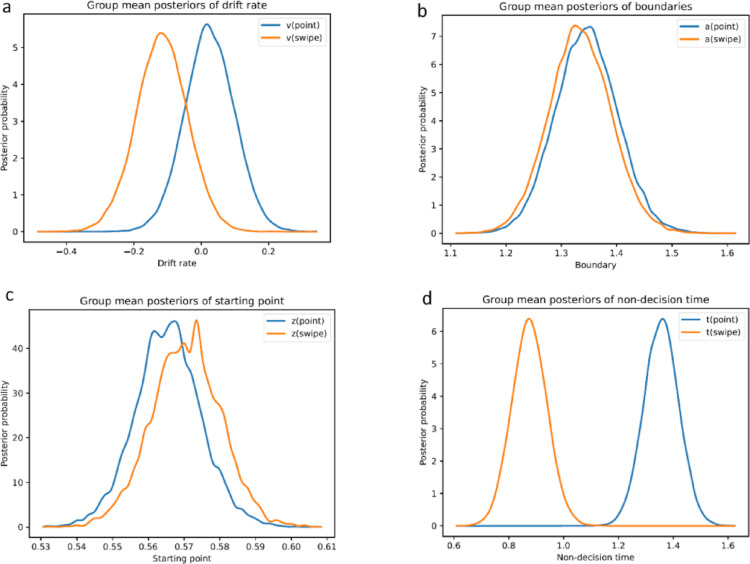
Posterior probabilities of within-subject DDM parameters based on response modality. *Note* Posterior probability distributions of pointing and swiping trials for the DDM parameters drift rate (**a**), boundary separation (**b**), starting point (**c**), and non-decision time (**d**)

The analysis of posterior distributions of HDDM parameters (Fig. 2) between both modalities indicated significantly shorter non-decision times for swiping compared to pointing, whereas no differences according to our posterior criterion emerged for drift rates, boundary separation and starting points (Table 2), although the posterior distribution of drift rates showed a descriptive tendency toward pointing (i.e., higher drift rate in pointing trials). When correlating trials between response modalities, significant positive correlations between drift rates, *r*(25) = 0.80, *p* < 0.001, and non-decision times, *r*(25) = 0.52, *p* = 0.021, of pointing and swiping trials are found.

**Table 2:** Descriptives of DDM parameters

DDM parameter	point		swipe		P_p|D_ [point > swipe]
	*M*	*SD*	*M*	*SD*	
v	0.02	0.24	− 0.12	0.23	.907
a	1.35	0.28	1.33	0.25	.560
z	0.26	0.03	0.29	0.04	.355
t	1.33	0.22	0.97	0.38	**1.00**

*v* Drift rate, *a* Boundary separation, *z* Starting point, *t* Non-decision time; ^1^Holm-Bonferroni corrected

Bolt numbers indicate significant results.

### Effects of response modality on tracking parameters

An investigation into the tracking parameters reveals that response modality has a significant effect on AUC, x-flips, velocity and entropy (Table 3). Pointing trials showed a greater AUC (i.e., a greater deviation from the direct movement path), a higher number of x-flips (i.e., more directional changes) and increased entropy (i.e., greater variability of the trajectory) as well as higher maximum velocity (i.e., faster peak movement execution) compared to swiping trials. In contrast, swiping trials were more direct and exhibited less variable trajectories. However, none of the parameters correlated across response modalities.

**Table 3:** Descriptives of tracking parameters

Tracking parameter	point		swipe		*t*	*df*	*p*^*1*^	*d*
	*M*	*SD*	*M*	*SD*				
AUC	0.30	0.14	0.21	0.09	2.86	26	.008	0.77
x-flips	37.06	13.9	1.09	0.43	13.51	26	< .001	3.66
vel	6.81	0.96	5.49	1.48	3.78	26	< .001	1.06
entropy	0.19	0.09	0.10	0.05	4.31	26	< .001	1.20

*AUC* Area under curve, *vel* Maximum velocity; ^1^Holm-Bonferroni corrected

### Mapping DDM to motion tracking parameters

To bridge cognitive modeling and motion tracking approaches, we correlated drift rate and non-decision time with all tracking parameters within each response modality (Table 4). After correcting for multiple analyses via Holm-Bonferroni correction, the results only showed a strong positive relationship between non-decision time and x-flips in pointing trials, indicating that the more x-flips, the longer the non-decision time.

**Table 4:** Correlations between HDDM and tracking parameters by response modality

HDDM	Tracking	pointing			swiping		
		*r*	*95%-CI*	*p*^*1*^	*r*	*95%-CI*	*p*^*1*^
v	AUC	.16	[− 1.0, .46]	> 1.0	.11	[− 1.0, .42]	> 1.0
v	x-flips	− .20	[− 1.0, .14]	> 1.0	.01	[− 1.0, .33]	> 1.0
v	vel	− .22	[− .51, 1.0]	> 1.0	− .09	[− .40, 1.0]	> 1.0
v	entropy	.19	[− 1.0, .48]	> 1.0	.05	[− 1.0, .36]	> 1.0
t	AUC	.06	[− .27, 1.0]	> 1.0	.35	[.04, 1.0]	.524
t	x-flips	.62	[.38, 1.0]	**.005**	.45	[.14, 1.0]	.155
t	vel	− .35	[− 1.0, − .03]	.513	− .29	[− 1.0, .04]	.921
t	entropy	− .08	[− .39, 1.0]	> 1.0	− .29	[− .56, 1.0]	.926
a	vel	− .20	[− 1.0, .13]	> 1.0	− .50	[− 1.0, − .21]	.065

*RT* Response time, *v* Drift rate, *a* Boundary separation, *t* Non-decision time, *z* Starting point, *AUC* Area under curve, *vel* Maximum velocity; ^1^tested one-sided and Holm-Bonferroni corrected

Bolt numbers indicate significant results.

Separate multiple linear regressions for each motion tracking parameter on the HDDM parameters showed a significant effect only for the motion tracking variable maximum velocity, *F*(4,22) = 2.91, *p* = 0.045, adj. *R*^2^ = 0.23, whereby only boundary separation showed a significant effect, *B* = − 1.65, *SE* = 0.71, *β* = 0.38, *t* = − 2.32, *p* = 0.030, indicating that higher boundary separation is related to a lower maximum velocity. The remaining models predicting AUC, x-flips, and entropy from HDDM parameters were non-significant. Separate analyses by response modality indicated a significant model for x-flips in pointing trials, *F*(4,22) = 5.25, *p* = 0.004, adj. *R*^2^ = 0.40. For details of individual predictors see Table 5.

**Table 5:** Regression of x-flips on HDDM parameters in pointing condition

Outcome	Predictor	B	SE	β	t	*p*	95%-CI
pointing							
x-flips	Intercept	− 31.68	23.37	0.00	− 1.36	.189	[− 80.15, 16.78]
	v	0.01	9.74	0.00	0.00	.999	[− 20.18, 20.21]
	a	16.01	8.06	0.32	1.99	.060	[− 0.71, 32.72]
	t	35.97	11.00	0.56	3.27	**.004**	[13.14, 58.79]
	z	− 3.06	88.61	− 0.01	− 0.04	.973	[− 186.83, 180.71]

*v* drift rate, *a* boundary separation, *t* non-decision time, *z* starting point

Bolt numbers indicate significant results.

## Discussion

In this study, we compared drift diffusion modeling (DDM) and finger tracking as measures of the time course of decision making in split-second handball penalty decisions. This allowed us to test whether both approaches yield similar insights into the processes underlying decision-making as suggested by their widespread use in existing research (e.g., Freeman 2018; Hehman, et al. 2015; Koul et al. 2019; Maldonado et al. 2019; Myers et al. 2022; Ratcliff et al. 2016). The current study adds to previous research (e.g., Leontyev and Yamauchi 2021; Weinberg et al. 2025) by modeling and comparing both DDM parameters as well as motion tracking indicators between two task modalities (i.e., pointing vs. swiping). Given that both modeling approaches promise to capture the underlying cognitive processes during decision making, we predicted individual model parameters to be related between tasks as the core decision process is typically assumed to be similar, that is, to be independent of the specific response movement.

### Relationship of DDM parameters between response modalities

Consequently, we predicted a positive correlation between DDM parameters across response modalities (e.g., drift rate in swiping should be positively correlated with drift rate in pointing). Whereas this positive relationship was confirmed for the DDM’s drift rate and non-decision time, no relationships were found for boundary separation and starting point. The positive correlation on non-decision time between response modalities might be driven by the fact that, as non-decision time represents both perceptual as well as motor response processes (Ratcliff and McKoon 2008), perceptual requirements are virtually identical between both tasks and relatively stable per individual (Myers et al. 2022). In addition, due to the different motor response requirements between tasks (i.e., pointing vs. swiping), we predicted that non-decision times differ significantly between response modalities. Replicating previous studies (Gomez et al. 2015; Weinberg et al. 2025), results indeed revealed those differences by indicating larger non-decision times in pointing compared to swiping trials and they were also directly linked to differences in response times. One aspect to note is that the video clips used in the current experiment were of different lengths, so the stimulus duration varies. However, identical video clips were used across both response modalities and therefore would affect both response modalities equally. Furthermore, all clips were temporally occluded at ball release, ensuring that decision-relevant information was aligned across trials. Thus, variability in stimulus duration primarily reflects the natural characteristics of sport-specific actions.

### Motion tracking is sensitive to response modality

Second, we hypothesized that if finger tracking reflects a robust measure of decision making over time, a positive correlation should emerge between tracking parameters across both response modalities. Contrary to this hypothesis, the analyses of movement tracking parameters, i.e., AUC, x-flips, velocity, and entropy, revealed no correlations across response modalities. That is surprising given the fact that these parameters are supposed to describe the evolution of a decision over time (Freeman et al. 2011), and thus theoretically should be largely unaffected by response modality. Therefore, the results raise the question to what degree movement tracking parameters are reliable measures of cognitive components across different task modalities. Furthermore, as AUC can be interpreted as a measure of uncertainty and decision conflict (Wulff et al. 2021) and x-flips and entropy are complexity indices mirroring trajectory disorder (Hehman et al. 2015; Wulff et al. 2021), their higher values in the pointing trials suggest that pointing trials are more cognitively challenging than swiping trials. In contrast, DDM results indicated no differences in cognitive task difficulty (i.e., no differences in drift rates) between both response modalities.

The absence of cross-modality correlations in tracking parameters has to be discussed in the context of the parameters’ nature and the task’s motor demands. On the one hand, trajectory-based measures reflect the development of a decision within the motor contexts and therefore capture a combination of perceptual, decisional and motor processes. The requirement of continuous contact by enforcing movement at the same time can potentially limit directional changes and characteristics of the trajectory independent of cognitive processes, whereas pointing movements allow for greater degrees of freedom. Hence, the lack of cross-modality might not necessarily indicate a lack of informational value but highlights that these measures are dependent on the response modality through which the decision is communicated. This is in contrast with how movement tracking is typically promoted in the literature (e.g., Maldonado et al. 2019), namely, as a “window into decision making” with a high degree of modality invariance. Consequently, commonly used parameters might not be modality-invariant measures of uncertainty or cognitive conflict as discussed in the literature (e.g., Wulff et al. 2021). On the other hand, DDM captures the evidence accumulation as a latent variable while capturing the modality-specification of the motor execution in the non-decision time. Those differences might be the reason why the DDM exhibits greater stability across response-modalities.

### DDM and motion tracking as measures of the same constructs?

As a third and final hypothesis, we predicted relationships between DDM and movement parameters, based on the assumption that if both capture the same decision-making process, their parameters should behave similarly and correlate irrespective of the response modality. However, results revealed that only in pointing trials the DDM’s non-decision time was positively correlated with motion tracking’s x-flips. That finding is partly contrary to our initial hypothesis as well as to the findings of Weinberg and colleagues (2025), who reported positive correlations between non-decision time, AUC and x-flips as well as negative correlations between non-decision time and velocity. For swiping trials, however, similar to the current study they also found no correlations between drift rates and tracking parameters. It is noteworthy that the experimental tasks in the current study and those in Weinberg et al. (2025) are identical (apart from the additional motion capture), and DDM parameters showed similar effects in both studies. Thus, the discrepant findings are likely driven by the motion tracking parameters which, as discussed previously, seem to be sensitive to changes in task modality. To investigate that further, we examined whether individual motion tracking parameters can be regressed on the set of DDM parameters to account for the fact that single tracking parameters might capture integrated components of the decision-making process. Those analyses indicated that boundary separation and non-decision time are correlated with subsets of tracking parameters while drift rate and starting point remain unrelated. Particularly non-decision time seems to be related to trajectory dynamics and complexity in a modality-dependent way. Those results emphasize the view that individual tracking parameters should not be interpreted as direct indicators of single decision components but rather as measures which reflect the interaction of cognitive and motor processes. In contrast, drift rate (i.e., evidence accumulation) proved to be robust across response modalities. From a theoretical perspective, the differences seen are consistent with the core assumptions underlying each methodological approach. On the one hand, DDM conceptualizes decision-making as a distinct evidence accumulation process with motor processes captured separately within the non-decision time (Ratcliff and McKoon 2008). Accordingly, the cognitive parameter drift rate is expected to be invariant across response modalities when the kinematic information remains identical. In contrast, movement tracking does not assume a distinction between cognitive and motor components and treats the trajectory itself as an expression of the developing decision (Wirth et al. 2020). Consequently, movement tracking inherently integrates perceptual, cognitive and motor processes and may therefore be more sensitive to specific constraints of the response modality. The results replicate findings of Weinberg and colleagues (2025) and support the applicability of DDM in controlled time-constraint anticipations tasks such as in the currently employed handball anticipation setting.

The present findings demonstrate that the single cognitive component of the decision-making process can be captured using DDM (e.g., Gomez et al. 2015; Leontyev and Yamauchi 2021), while finger tracking reflects an integrated decision-action process and therefore seems to be more strongly shaped by movement-specific constraints in the present task. In fact, the current results raise the question to what extent motion tracking is able to represent the underlying decision process. It is well known that tracking parameters can be influenced by the experimental task (Wirth et al. 2020). As such, it is conceivable that the task modality affected the tracking outcomes: In the pointing condition, after initiating a trial by touch participants were able to move their finger without spatial or temporal restrictions. However, in the swiping condition, uninterrupted contact with the tablet surface as well as continuous forward movements were mandatory. The specific constraints of the response modality result in different demands on movement initiation and dynamics as well as online control, even if the movement endpoint is similar. Those differences can also be seen in the descriptive measures, such as longer overall response times, larger AUC, more x-flips, higher entropy and higher maximum velocity in pointing compared to swiping trials. Those different task demands might have influenced not only the monitored movement but also have impacted movement control (Olthuis et al. 2020). Whereas the DDM captures those task differences separately in the non-decision time, the tracking parameters could not isolate those from the cognitive demands. Furthermore, execution variability due to body and movement adjustments (Cowin et al. 2022) within the response modalities could lead to fundamental differences in trajectories and high deviations in trajectory measurements. These known influences of the behavioral context of the task may interfere with the ability of motion tracking parameters to capture the cognitive basis of the decision-making process. This might also explain why correlations between DDM parameters and conceptually related parameters of motion tracking were inconsistent.

To evaluate whether the reduction from 3D to 2D might have influenced the tracking parameters, we examined the spatial variance of the trajectories. The majority of the total movement variance was located within the tablet-aligned x–y dimension (97.8%), and the variability along the discarded z-axis was not systematically related to the x–y deviation, suggesting that the dimensionality reduction is unlikely to account for the observed differences in response modality in the tracking parameters. However, future research could extend the current approach by incorporating full 3D trajectories to explore whether additional spatial information provides further insight into decision-making in more complex settings. However, the data of the current study are also compatible with the idea that both methods capture different aspects of decision-making. On the one hand, DDM models the unfolding of perception, decision-making, and motor response in a sequential approach where evidence accumulation precedes action. On the other hand, movement tracking may rather reflect an aggregate of the integrated dynamics of perception, evidence accumulation, and action. Consequently, it may be more sensitive to movement-specific constraints. Note though, that such an approach is also compatible with recent discussions in the DDM literature proposing a continuous interaction between evidence accumulation and motor processes (Dendauw et al. 2024; Servant et al. 2021). Finally, the differences between both approaches might highlight their joint value when investigating decision-making: Whereas the DDM captures the decision-making process aside motor constraints, movement tracking provides access to how evidence accumulation and motor processes are coupled during anticipatory behavior.

A limitation of the current study concerns the employed response modalities. While pointing and swiping enabled us to investigate decision-making in a controlled environment, they cannot mirror the whole-body responses required in real sport situations. Consequently, the present findings should be interpreted as a first step toward examining decision-making in a more naturalistic setting before extending those approaches to more complex environments. Notably, even comparatively small variations in response modality were sufficient to affect motion tracking parameters, suggesting that such measures might be sensitive to motor demands. In contrast, decision-related DDM parameters appeared robust across the response modalities within our decision-making task, which indicates a relative independence from motor execution constraints. However, it might be the case that also DDM parameters are affected by response modality if the difference between modalities is amplified. Future research should therefore examine a broader range of response modalities to potentially identify which movement types permit a clearer distinction of cognitive (i.e., decision-making) components. The impact of task-specific movements is particularly relevant in the context of anticipation and therefore should be considered in experimental settings (Nakayama et al. 2023; Michalski et al. 2020). Our decision task incorporated only two-dimensional video stimuli, which lack stimuli and constraints present in real-world settings (for a critical review, see Johnson 2025) but allowed for experimental control. Therefore, future research should extend anticipation tasks to more complex and interactive settings (see also Cañal-Bruland and Mann 2025), including whole-body response measures. Based on the substantial effects of rather minimal changes in response modality (pointing vs. swiping) in the current task it may very well be that motion tracking parameters are even more volatile if one adopts more ecologically valid response measures.

## Conclusion

Taken together, our findings demonstrate that DDM reflects the decision-making process in a time-pressured anticipation task such as a handball penalty. Building upon existing research, we employed an optical motion capture system to trace trajectories of both pointing and swiping movements and calculated drift–diffusion models grouped by those response modalities. The DDM captured movement-related differences between pointing and swiping in the non-decision time, as indicated by consistent correlations between response modalities. The movement tracking parameters (i.e., AUC, maximum velocity, x-flips, and entropy), however, did not show consistent correlations between pointing and swiping trials. The results suggest that tracking parameters are tied to the response modality through which the decision is expressed and therefore reflect a modality-specific integrated process of the decision-making process. In contrast, by separating the evidence accumulation from the motor execution, DDM appears well suited for isolating the decision-related processes across response modalities, while movement tracking may be more informative for examining the continuous coupling of decisional and motor processes.

## Data Availability

The data will be shared in an open repository upon acceptance for publication.

## Footnotes

1 The participants were included in all analyses. Expertise-related effects were not analyzed.

2 The lowest ESS and highest R-hat were observed for z(point), i.e., the starting point in the pointing condition: ESS_bulk = 398.74, R-hat = 1.0160, likely reflecting the absence of a bias manipulation and thus limited the identifiability of starting point. However, the deviation from the recommended ESS criterion was marginal, accompanied by a stable R-hat value (R-hat < 1.05; Pan et al. 2025), and did not affect the overall pattern of results.
